# Supplementary material for: Over-The-Counter Codeine: Can Community Pharmacy Staff Nudge Customers into Its Safe and Appropriate Use?
Source: Pharmacy (Basel). 2020 Oct 8;8(4):185. doi: 10.3390/pharmacy8040185 (PMC7712583; doi:10.3390/pharmacy8040185)
Supplement: Supplementary file 1 [file pharmacy-08-00185-s001.pdf]

pharmacy

MDPI

Supplementary Materials: The following are available online at www.mdpi.com/xxx/s1. Figure S1: Boots Patient Safety Card; Figure S2: Data tracker; Table S1: Survey questions for pharmacy teams.

Figure S1. Boots Patient Safety Card

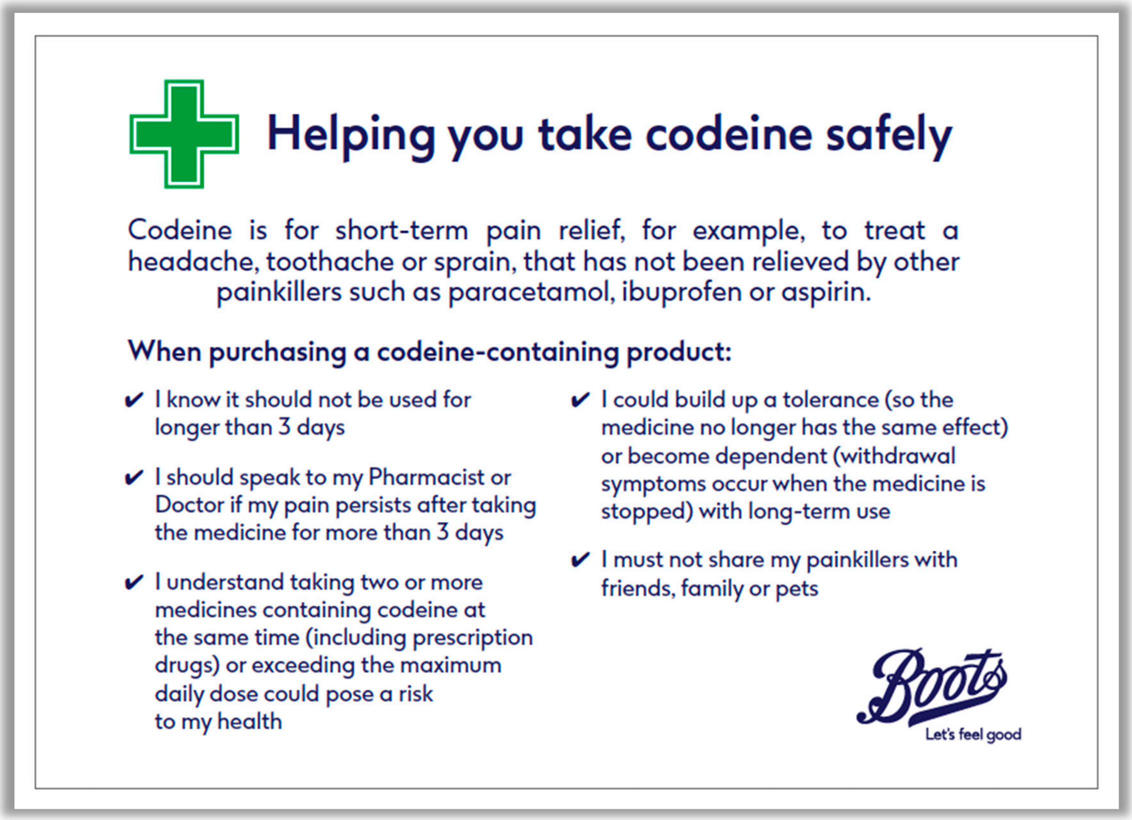

Figure S2. Data tracker.

Codeine Patient Safety Card
Please complete this form for all sales of codeine containing medicines

Date	How easy / difficult did you find it to initiate the conversation about the Card?<br><br>1: very easy<br>2: quite easy<br>3: quite difficult<br>4: very difficult	How receptive was the customer to engage with you about the Card?<br><br>1: very receptive<br>2: quite receptive<br>3. not receptive<br>4: refused the Card	Outcome of Customer Interaction - tick one only					Tick where applicable		Any other comments or notes?
			Customer purchased **original codeine containing medicine**	Customer purchased **smaller pack of codeine containing medicine** (if available)	Customer purchased alternative **non-codeine containing medicine or product**	Customer did **not make a purchase** for their pain relief	Customer did **not make a purchase** for their pain relief **AND signposted**	Customer **asked a pharmacy team member questions** about codeine or pain management	This is a **known customer that makes frequent codeine purchases**	

Store Number: **Sheet Number:**

Table S1. Survey questions for pharmacy teams.

1. Please confirm your job role:
    - Store Manager
    - Pharmacist Store Manager
    - Pharmacist
    - Pre-reg pharmacist
    - Accuracy Checking Pharmacy Technician
    - Trainee Accuracy Checking Pharmacy Technician
    - Pharmacy Technician
    - Trainee Pharmacy Technician
    - Pharmacy Advisor
    - Trainee Pharmacy Advisor
    - Other (please specify)
2. Please enter your store number:
3. Have you used the Patient Safety Card when selling OTC codeine-containing medicines?
    - Yes
    - No
4. Did the Patient Safety Card support your conversations with customers about the safe and appropriate use of OTC codeine?
    - Yes
    - No
5. Please explain your response to the previous question (Question 4).
6. Do you consider using the Patient Safety Card helped to achieve the most appropriate outcome for the customer (for example, sale of the most appropriate product or signposting)?
    - Yes
    - No
7. Please explain your response to the previous question (Question 6).
8. Please describe any barriers or operational issues with the Patient Safety Card?
9. Would you recommend using the Patient Safety Card to other Pharmacy Teams?
    - Yes
    - Maybe
    - No
    - Don't know
10. Please explain your response to the previous question (Question 9).

11. Please provide any feedback on how the text or format of the Patient Safety Card could be improved?
12. Is there further training or support that could improve conversations with customers about OTC codeine? Select all that apply.

- Clinical expertise, for example, in pain management, specialist addiction training or harm reduction training
- Professional matters, for example, how to deliver counselling and advice if abuse/misuse if suspected or how to best signpost customers to another healthcare provider
- Soft skills, for example, supporting customers with behaviour change, reading body language or communication skills
- No further training or support required
- Other (please specify)

13. Do you think any other measures should be implemented to support the safe and appropriate supply of OTC codeine-containing medicines? Select all that apply.

- Patient education and awareness campaign
- Provide additional patient information at the point of sale (such as the Patient Safety Card)
- Require every sale to involve a consultation with the pharmacist
- Cease all promotional activity including advertising
- Reduce the pack sizes
- Pharmacist-led pain management service
- Keep records of sales (national database) and signpost as appropriate
- Switch to Prescription Only Medicine
- Other (please specify)

***

If No to Q3:
4. Please describe any barriers or operational issues with the Patient Safety Card?
5. Would you recommend using the Patient Safety Card to other Pharmacy Teams?

- Yes
- Maybe
- No
- Don't know

6. Please explain your response to the previous question.
7. Please provide any feedback on how the text or format of the Patient Safety Card could be improved?
8. Is there further training or support that could improve conversations with customers about OTC codeine? Select all that apply.

- Clinical expertise, for example, in pain management, specialist addiction training or harm reduction training
- Professional matters, for example, how to deliver counselling and advice if abuse/misuse if suspected or how to best signpost customers to another healthcare provider
- Soft skills, for example, supporting customers with behaviour change, reading body language or communication skills
- No further training or support required
- Other (please specify)

9. Do you think any other measures should be implemented to support the safe and appropriate supply of OTC codeine-containing medicines? Select all that apply.

- Patient education and awareness campaign
- Provide additional patient information at the point of sale (such as the Patient Safety Card)
- Require every sale to involve a consultation with the pharmacist
- Cease all promotional activity including advertising
- Reduce the pack sizes
- Pharmacist-led pain management service
- Keep records of sales (national database) and signpost as appropriate

Switch to Prescription Only
